# Non-Extensive Statistical Analysis of Acoustic Emissions: The Variability of Entropic Index q during Loading of Brittle Materials Until Fracture

**DOI:** 10.3390/e23030276

**Published:** 2021-02-25

**Authors:** Andronikos Loukidis, Dimos Triantis, Ilias Stavrakas

**Affiliations:** Electronic Devices and Materials Laboratory, Department of Electrical and Electronics Engineering, University of West Attica, 250 Thivon Avenue, 122 44 Athens, Greece; triantis@uniwa.gr (D.T.); ilias@uniwa.gr (I.S.)

**Keywords:** non-extensive statistical mechanics, Tsallis entropy, q-exponential function, acoustic emissions, brittle materials

## Abstract

Non-extensive statistical mechanics (NESM), introduced by Tsallis based on the principle of non-additive entropy, is a generalisation of the Boltzmann–Gibbs statistics. NESM has been shown to provide the necessary theoretical and analytical implementation for studying complex systems such as the fracture mechanisms and crack evolution processes that occur in mechanically loaded specimens of brittle materials. In the current work, acoustic emission (AE) data recorded when marble and cement mortar specimens were subjected to three distinct loading protocols until fracture, are discussed in the context of NESM. The NESM analysis showed that the cumulative distribution functions of the AE interevent times (i.e., the time interval between successive AE hits) follow a q-exponential function. For each examined specimen, the corresponding Tsallis entropic q-indices and the parameters *β_q_* and $\tau_{q}$ were calculated. The entropic index q shows a systematic behaviour strongly related to the various stages of the implemented loading protocols for all the examined specimens. Results seem to support the idea of using the entropic index q as a potential pre-failure indicator for the impending catastrophic fracture of the mechanically loaded specimens.

## 1. Introduction

In the quest to understand the latent mechanisms of fracture and crack development in stressed materials, many non-destructive monitoring techniques have been used. Amongst them is the acoustic emission (AE) technique, which is based on the detection of the transient elastic waves that are produced due to the nucleation and propagation of cracks. Considering isotropic, linear materials, these waves propagate inside the material in a spherical manner towards its surface, where they are detected by properly attached piezoelectric sensors. The study of AE can provide information regarding the fracture mechanisms as well as the crack generation and development processes taking place inside the mechanically stressed materials [1]. Several AE parameters and indices have been considered as potential pre-failure indicators for the estimation of the upcoming catastrophic fractures [2,3,4,5]. The AE technique has been employed successfully in monitoring the overall accumulated damage and remaining service life in situ (e.g., industrial equipment, full-scale structures, ancient monuments) and laboratory scale (e.g., mechanically loaded specimens) [6,7,8,9].

Taking into account that the AEs are considered to be a form of microseismicity, it is reasonable to assume that tools used for the analysis of earthquakes could also be used to analyse the AE activity after proper modifications [10,11,12,13]. In this direction, assuming that the fracture phenomena which instigate the creation of both earthquakes and AEs, although at different scales, are non-linear procedures of intricate dynamical systems during their non-equilibrium stationary states—one may even consider them as phase transitions—are governed by multi-fractality, self-similar structure, exhibiting large scale correlations and memory effects; the use of advance statistical tools is advocated, instead of the classical statistical physics [14,15,16,17,18,19,20]. Non-extensive statistical mechanics (NESM) introduced by Tsallis [21,22,23,24,25], based on the principle of entropy have been proven a reliable statistical framework for analysing intricate dynamical systems such as the fracture mechanisms responsible for the generation of earthquakes and AEs. NESM has been applied in a variety of fields [23]; here, the authors focus mainly on NESM applications of seismology [26,27,28,29], plate tectonics [30,31,32,33,34,35] and fracture mechanics [36,37,38,39,40,41,42]. Taking into account the similarities between seismicity and AE activity, as already mentioned, the application of NESM in fracture mechanics concerning stressed rocks is valid. As such, several works have been conducted, attempting to study the fracture processes occurring in mechanically loaded brittle materials (e.g., rocks, cement mortar) through the recorded AEs under the scope of NESM [36,37,38,39,40,41,42]. Vallianatos et al. [36] studied the recorded AE activity in terms of NESM, when basalt specimens were subjected to diametral compression until fracture. Results showed that the cumulative distributions of the AEs’ scalar moment, the cumulative distributions of the three-dimensional distances (i.e., the Euclidean distances) between successive AE events and the time-intervals between successive AE events (i.e., the AE inter-event times) obey q-exponential distributions. Stergiopoulos et al. [37] showed that, under the concept of NESM, the cumulative distributions of the time-intervals between successive AE events recorded from cement mortar specimens, made of ordinary Portland cement, when subjected to three-point bending, are characterised by q-exponential functions and the calculated entropic q-indices are strongly correlated to the applied mechanical load. Stavrakas et al. [38] studied the AE events recorded from cement mortar specimens, made of white cement, when subjected to six repetitive loading–unloading loops of three-point bending. The recorded AE data were analysed under NESM, showing that the cumulative distributions of the time-intervals between successive AE events follow a q-exponential function, and that the calculated q entropic indices, as well as the relaxation parameters $\beta_{q}$, present systematic changes throughout the duration of the individual loading–unloading loops of the loading protocol. In the study by Saltas et al. [40], sandstone and marble specimens were subjected to diametral compression until fracture, while the AE activity was recorded. The recorded AE data were analysed under the NESM framework. The analysis showed that the cumulative distributions of the inter-event times between successive AE events groups, for both kinds of specimens, obey a q-exponential function. In addition, the authors studied the temporal evolution of the entropic q-indices and the corresponding relaxation parameters $\beta_{q}$, of both classes of specimens, and as functions of the applied mechanical load. Results showed that the calculated entropic q-indices and the relaxation parameters $\beta_{q}$ present a strong relationship to the applied mechanical load. Greco et al. [41] analysed the AE data recorded during cyclic compression tests of concrete and basalt specimens through NESM. The cumulative distributions of the AE inter-event times showed that they follow a q-exponential form with the q entropic indices and the $\beta_{q}$ relaxation parameters exhibiting distinct changes during the different stages of the conducted experiment.

The purpose of the present work is to utilise the AE timeseries obtained when specimens of various geometries made of brittle materials i.e., Greek Dionysos marble and cement-mortar, made of ordinary Portland Cement, which were subjected to various loading protocols until fracture, and then analysed using NESM. The first step was to determine whether the cumulative distribution of the time intervals in successive AE hit groups until the fracture of the specimens, obeys a q-exponential form. In the second phase, after extracting the values of the Tsallis entropic index q, the aim was to determine if there was systematic variability of q, in the various stages of the sample loading protocol until they are broken. The latter can provide valuable information about the impending fracture.

## 2. Theoretical Background

In order to describe multifractal and self-similar systems, with long range interactions and memory effects, Tsallis introduced a generalisation of the Boltzmann–Gibbs (BG) statistics [21]. The formulation of this generalised entropy $S_{q}$, called Tsallis entropy, which in the case of a variable X with probability distribution function (PDF) p(X) is defined as [21,22,23,24,25]:

(1) $$S_{q}=k_{B}\frac{1}{q-1}\left(1-\sum_{i=1}^{w}p_{i}^{q}\right)$$

where $k_{B}$ is Boltzmann’s constant, w are the number of the total known microstates of the physical system, $p_{i}$ are the probabilities associated with the microstates w, and q is the entropic index. The latter expresses the degree of non-additivity of the physical system [21,22,23,24,25]. The ordinary BG entropy formulation is obtained for q→1: $S_{BG}=-k_{B}\sum_{i=1}^{w}p_{i}\ln p_{i}$; *q* > 1 leads to sub-additivity, and q<1 to super-additivity.

Tsallis entropy ($S_{q}$) as a generalised approach shares many common properties with the standard BG entropy ($S_{BG}$), such as positivity, concavity, Lesche stability, and extremization for the uniform distribution (for more common properties see Table 3.10 of ref [24]). The main difference between BG and Tsallis entropy is the fact that BG entropy $S_{BG}$ is additive while Tsallis entropy $S_{q}$ is non additive. BG entropy $S_{BG}$, exhibits short-range correlations and the total entropy depends on the size of the subsystems and the total microstates comprising the physical system. Tsallis entropy $S_{q}$ (for q≠1) is non-additive, i.e., if two identical subsystems combine, the entropy of the resultant system is not equal to the summation entropy of its subsystems (Equation (2)). Furthermore, Tsallis entropy $S_{q}$ exhibits long range correlations and appears to be more suitable for complex dynamical systems [21].

A physical system consists of two probabilistically independent sub-systems, namely, *A* and B. The additive aspect of the BG entropy is violated, thus the Tsallis entropy $S_{q}$ satisfies Equation (2), which describes the non-additive behaviour of the physical system and is referred to as pseudoadditivity:

(2) $$S_{q}\left(A+B\right)=S_{q}\left(A\right)+S_{q}\left(B\right)+\frac{1-q}{k_{B}}S_{q}\left(A\right)S_{q}\left(B\right)$$

The rightmost term of Equation (2) indicates the interplay amongst the two subsystems and constitutes the origin of non-additivity of the physical system (i.e., in our case the mechanically active specimens). In addition, it manifests the dependency of long-range interactions between the fracture evolution processes caused by the AE events. For a super-additive system based on Equation (2), we have $S_{q}\left(A+B\right)>S_{q}\left(A\right)+S_{q}\left(B\right)$ and for a sub-additive system $S_{q}\left(A+B\right)<S_{q}\left(A\right)+S_{q}\left(B\right)$ [23].

The probability distribution p(X) of the acoustic parameter X is attained through the maximisation of the non-extensive Tsallis entropy by introducing at least two appropriate constraints (i.e., the normalisation condition p(X) and the generalised q-expectation value *X_q_*), using the Lagrange-multipliers method [21,24]. The normalisation of the distribution p(X) is: $\int_{0}^{\infty }p\left(X\right)dX=1$ and the q-expectation value, $X_{q}$, is given according to the formula: $X_{q}=\langle X_{q}\rangle =\int_{0}^{\infty }XP_{q}\left(X\right)dX=1$, with $P_{q}\left(X\right)$ being the escort probability that is given by [24]:

(3) $$P_{q}\left(X\right)=\frac{P^{q}\left(X\right)}{\int_{0}^{\infty }P^{q}\left(X\right)dX^{\prime }}$$

The maximisation of Tsallis entropy $S_{q}$, leads to the following optimal probability density function [21,24]:

(4) $$p\left(X\right)=\frac{1}{Z_{q}}{\left[1-(1-q)\beta_{q}X\right]}^{1/(1-q)}=\frac{1}{Z_{q}}\exp_{q}(-\beta_{q}X)$$

where $Z_{q}$ is the q partition function: $Z_{q}=\int_{0}^{X\max}\exp_{q}\left(-\beta_{q}X\right)dX$. The entropic parameter $\beta_{q}$ is defined as: $\beta_{q}=\beta^{*}/\left(c_{q}+\left(1-q\right)\beta X_{q}\right)$, where *β^∗^* is the Lagrange multiplier and $c_{q}={\int_{0}^{X_{\max}}\left[p\left(X\right)\right]}^{q}dX$.

The term $\exp_{q}\left(X\right)$ signifies the “q-exponential function”, defined as:

(5) $$\begin{array}{cc} \exp_{q}\left(X\right)={\left[1+\left(1-q\right)X\right]}^{\frac{1}{1-q}} & \begin{array}{cc} \mathrm{when} & \left[1+\left(1-q\right)X\right]\geq 0 \end{array}\\ \exp_{q}\left(X\right)=0 & \begin{array}{cc} \mathrm{when} & \left[1+\left(1-q\right)X\right]<0 \end{array} \end{array}$$

The inverse is the “q-logarithmic function”:

(6) $$\ln_{q}\left(X\right)=\frac{1}{1-q}\left(X^{1-q}-1\right)$$

It is noted that for the case of BG statistics, when q→1, both Equations (5) and (6) converge to the ordinary exponential and logarithmic function, respectively. In the case of sub-additivity when q>1, a power law tail appears, whereas in the case of super-additivity when 0<q<1 the q-exponential function presents a cut-off [26,27].

According to refs [36,37,38,39,40,41,42], the quantity that should be compared with the distribution the system under study is not the original p(X), but its associated escort distribution $P_{q}\left(X\right)$. The normalised cumulative distribution of the acoustic parameter X, manifested as a q-exponential function, is obtained by integrating the probability density function p(X):

(7) $$P\left(>X\right)=\int_{X}^{\infty }P_{q}\left(X\right)dX=\exp_{q}\left(-\frac{1}{\left(1-q\right)\langle X_{q}\rangle +\frac{1}{\beta^{*}}}X\right)$$

A close inspection of the rightmost term of Equation (7) reveals that after the approximation of the suitable Tsallis entropic index q which describes the distribution of the acoustic parameter X, the logarithmic function lnq[P(>X)], which is calculated by basic algebra rules (i.e., $\ln_{q}\left[P\left(>X\right)\right]=a^{-1}X$), is linear in accordance to X with slope $a=-1/\left(1-q\right)\langle X_{q}\rangle +\frac{1}{\beta^{*}}$.

**Figure 1:**
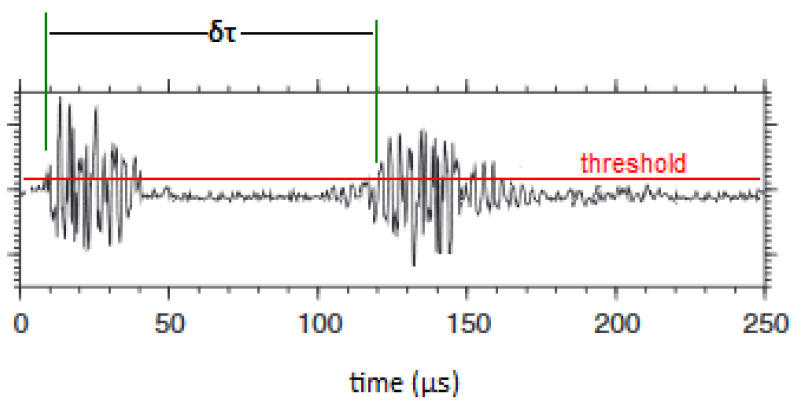
The method for calculating the interevent time δτ in the case of two consecutive AE hits.

For the needs of the present work, the continuous variable X represents the inter-event time *δτ* between the average occurrence time of successive AE hit groups. Thus, the original AE timeseries after its division to successive groups of adequate number of AE hits, is transformed to the inter-event time timeseries *δτ_i_* = *t*_*i*+1_ − *t_i_*. Figure 1 is indicative for the method used in calculating the interevent time δτ, i.e., the duration between successive hits, which expresses the time interval between the beginning of one hit and the beginning of the next. It should be noted that the user-defined threshold for the needs of the presented experiments was set to 40bB. Subsequently, NESM was applied to the inter-event times timeseries $\delta \tau_{i}$, and the normalised cumulative distribution of the inter-event times timeseries P(>δτ), was plotted for each AE hit group. The AE data were fitted with a q-exponential function and the entropic indices q with the relaxation parameter $\beta_{q}=1/\tau_{q}$, referring to each AE hit group, were calculated.

## 3. Specimens

Four experiments are presented here involving three Greek Dionysos marble specimens and one cement mortar specimen, based on ordinary Portland cement. In total, three loading protocols were implemented, during which the specimens fractured: (i) diametral compression; (ii) three-point bending; and (iii) direct tension. During all the experiments, the AE activity was recorded simultaneously. Greek Dionysos marble is used extensively for the restoration project of the temples upon the Athenian Acropolis because it presents the same physiochemical properties as Pentelic marble, the original building stone of the temples [8,43,44,45]. Cementitious materials, made of ordinary Portland cement, are the most common type of masonry mortar currently used in the construction industry [46], and therefore an early assessment of its mechanical status state is important for estimating its remaining loading carrying capacity. Table 1 summarises the type of the implemented loading protocols, alongside the materials and the total number of the AE hits that were recorded during the each presented experiment. Only essential information will be presented here; thus, detailed descriptions of the conducted experiments and the experimental set-up used can be found in the corresponding references, which will be indicated later in the text.

The experiment that will be referred to herein as EXP-1 was conducted on a beam-shaped marble specimen with a square cross-section. The dimensions of the specimen were 20 × 20 × 100 mm^3^ and the notch dimensions were 2.5 mm in width and 4 mm in length. The specimen was subjected to three-point bending under displacement-control mode at a rate of 0.01 mm/min [47]. So-called EXP-2 was conducted on a marble double-edge notched specimen of dog-bone shape. The thickness of the specimen was 1.2 cm, and the length of the notches was equal to 4 cm. The specimen was subjected to direct tension under displacement-control mode at a rate of 0.2 mm/min [48]. EXP-3 herein refers to an experiment that was conducted on a marble specimen of a circular semi-ring (CSR) shape, based on the geometry proposed in ref [49], with outer diameter equal to 100 mm and inner diameter equal to 50 mm. The CSR specimen was subjected to diametral compression under displacement-control mode at a rate equal to 0.02 mm/min [50]. A beamed-shaped cement mortar specimen that was prepared according to the details described in ref [51] was subjected to a three-point bending loading up to fracture. This experiment will herein be referred to as EXP-4. The beamed-shaped specimen’s dimensions were 50 × 50 × 200 mm^3^. The specimen was subjected to three-point bending under constant loading rate of approximately 35 N/s [51].

**Figure 2:**
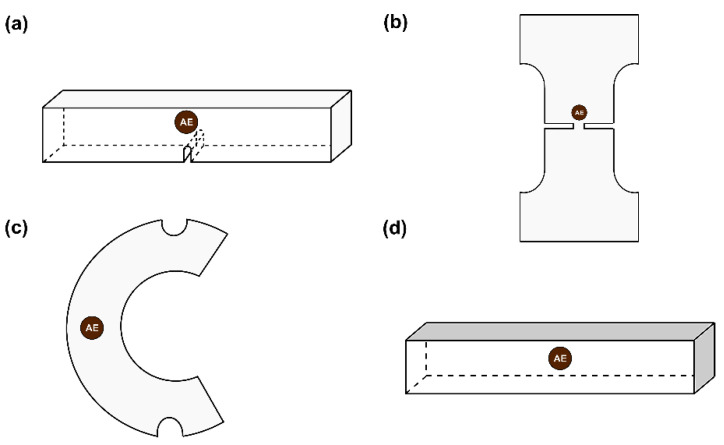
Basic geometries of the presented specimens and the position of the acoustic sensor considered in the present study (brown circles): (**a**) centrally notched marble specimen EXP-1; (**b**) dog-bone shaped marble specimen EXP-2; (**c**) circular semi-ring marble specimen EXP-3; and (**d**) cement mortal specimen EXP-4. Note that the sketches are not to scale.

All the experiments were conducted using an electromechanical electromagnetic MTS-Insight loading frame of 10kN loading capacity. In order to detect and monitor the AE hits, R15α acoustic sensors were attached to each specimen. Specifically, in the case of EXP-1, four (4) R15α acoustic sensors were deployed; for EXP-2 six (6) R15α acoustic sensors were placed on the specimen; in the case of EXP-3 three (3) R15α sensors; and during EXP-4 one (1) R15a sensor was mounted in the middle of the specimen’s surface. The AE data presented here originate from the AE sensor closer to the fracture (Figure 2).

## 4. Results and Discussion

The formulation of the normalised cumulative distribution function (CDF) of the AE inter-event times P(>δτ), based on Equation (7), obeys a q-exponential distribution proposed by ref [36] for AE data recorded from basalt specimens subjected to mechanical loading until fracture and verified in the cases of AE data recorded from mechanically loaded marble, sandstone and cement mortar specimens [37,38,39,40,41,42]:

(8) $$P\left(>\delta \tau \right)=\exp_{q}\left(\beta_{q}\cdot \delta \tau \right)={\left[1+\left(q-1\right)\beta_{q}\cdot \delta \tau \right]}^{\frac{1}{1-q}}$$

where $\beta_{q}=1/\tau_{q}$ is an entropic parameter with inverse time dimensions. The parameter $\tau_{q}$ is a time parameter associated with the average value $\bar{\delta \tau }$ of the AE inter-event times [40]:

(9) $$\frac{\bar{\delta \tau }}{\tau_{q}}=\frac{B\left(2,\frac{2-q}{q-1}\right)}{{\left(q-1\right)}^{2}}$$

The expression $\bar{\delta \tau }/\tau_{q}$ has been found to obey a distribution dictated by the Beta function [40,52]: $B\left(x,y\right)={\int_{0}^{1}t^{x-1}\left(1-t\right)}^{y-1}dt$. It is noted that the probability density function P(>X), given by Equation (7), and the normalised cumulative distribution function of the inter-event times of the average occurrence time of the AE hit groups P(>δτ), expressed by Equation (8), exhibit the same mathematical form.

In order to monitor the development and variation of the q and $\beta_{q}$ parameters, the AE hits that were recorded during each loading protocol were divided in k successive groups depending on the total number of the recorded AE hits. In order to make the analysis more reliable, the hits comprising each group should be a sufficient number, which by direct experimentation was estimated to be above 80 AE hits. The entropic q-index and the $\beta_{q}$ parameter of the q-exponential distributions were calculated for each fitting curve and are depicted as a function of the “average time before failure” parameter $\left(t_{f}-t_{k}\right)$, with $t_{f}$ being the moment of the failure of the specimen and $t_{k}$ as the average value of the occurrence time of the AE hits comprising each *k*th group. In the same figure, the normalised average value of the imposed load during the recording of each AE group, denoted as $\ell_{k}$, is also depicted. Table 1, Table 2, Table 3 and Table 4, summarise the calculated values of the q entropic indices, the values of the corresponding $\beta_{q}$ and $\tau_{q}$ parameters along with the normalised load values $\ell_{k}$, and the “average time before failure” parameter $\left(t_{f}-t_{k}\right)$.

**Figure 3:**
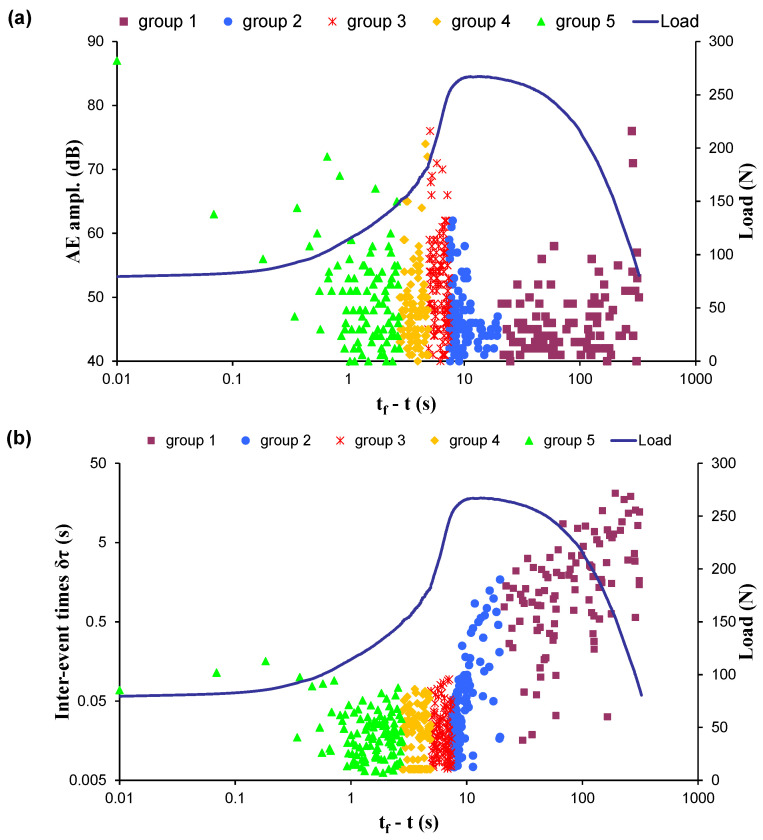
The distribution of (**a**) the AE amplitudes and (**b**) the inter-event times of the specimen EXP-3 in combination with the applied mechanical load in the “time to failure” $\left(t_{f}-t\right)$ time scale. Each colour signifies the different AE hit group used for the calculation of the corresponding inter-event times.

Subsequently, an indicative AE timeseries recorded during one of the four experiments studied in the present work is presented (Figure 3a)—specifically, for the case of the marble specimen EXP-3 which was subjected to diametral compression. Figure 3a shows the time recording of the AE hit amplitudes. In the same figure, the time evolution of the applied mechanical load is shown using the “time to failure” $\left(t_{f}-t\right)$ parameter as a time scale, in order to make the time distribution of the AEs more visible, especially during the last seconds of the loading protocol when the fracture of the specimen happens. In the specific experiment, the fracture of the specimen occurred at the moment $t_{f}=486.34s$, while the first AE hit was recorded for *t* = 162*s* $\left[t_{f}-t\approx 324s\right]$ when the applied load had attained an approximate value of L=80.5N. It was observed that the applied mechanical load reached its maximum value $L_{m}\approx 267N$ for $t=t_{m}\approx 473s$ $\left[t_{f}-t\approx 13s\right]$. Considering that until the failure of the specimen N=507 AE hits were recorded (506 inter-event times), the AE hits were organised in k=5 groups with n=100 consecutive inter-event times. Note that the last AE hit group included 105 inter-event times. Each AE hit group is shown using different colours. Figure 3b shows the distribution of the inter-event times of the AE hit groups in combination with the applied mechanical load in “time to failure” $\left(t_{f}-t\right)$, using the same colour format as in Figure 3a.

**Figure 4:**
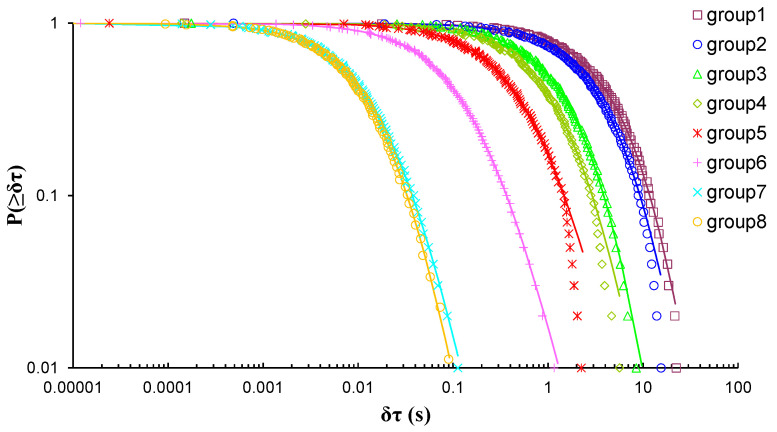
The cumulative distribution functions (CDFs) of the AE inter-event times of each AE hit group (circle markers) for EXP-1, along with the corresponding q-exponential fitting curves (solid curves).

In the case of EXP-1 which involved a marble beam-shaped specimen, due to the number of the recorded AE hits during the three-point bending loading protocol, eight groups of AE hits were formed, thus leading to the calculation of eight q-indices, each of them corresponding to a different loading stage of the loading process. Figure 4 shows the log–log plot of the cumulative distributions of the AE inter-event times *P*(>*δτ*) of the eight AE hit groups of EXP-1, while Table 2 present the corresponding q-indices and the fitting parameters $\beta_{q}$ and $\tau_{q}$, which have been calculated using Equation (8). Regarding the long times δτ of Figure 4 of all the depicted CDFs, especially those belonging to groups 2 to 5, it is evident that the fitting points diverge from the corresponding modelled ones, a behaviour which potentially could be regarded to the degree of selected subsystems and the limitation of the modelling. As such, with the intention of avoiding potential bias during the estimations of the Tsallis q-indices, the tail regions of the CDFs are excluded; thus, solely keeping the data points located at the initial and the central regions of the CDFs.

**Figure 5:**
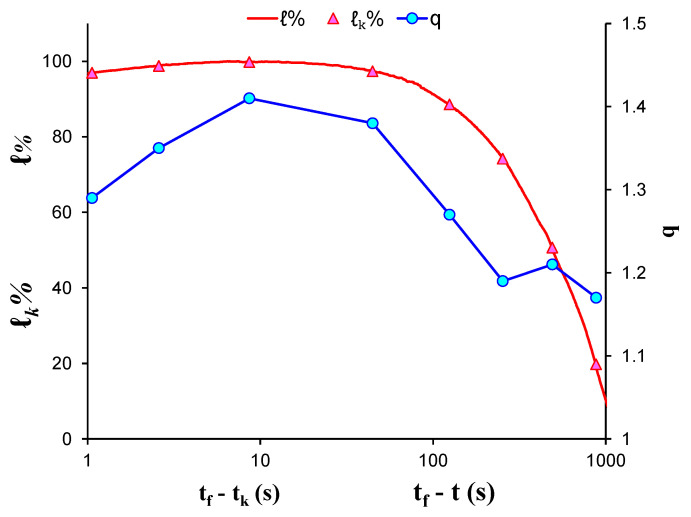
The variability of the entropic index q against the normalised average applied load $\ell_{k}$ for each AE hit group of EXP-1 in terms of the “average time before failure” parameter $\left(t_{f}-t_{k}\right)$ and the normalised applied load ℓ in “time to failure” $\left(t_{f}-t\right)$ time scale.

Figure 5 shows the temporal evolution of the calculated q indices alongside the normalised average applied load $\ell_{k}$ during the recording of each AE hit group using the “average time before failure” parameter $\left(t_{f}-t_{k}\right)$ for the time scale, with *t_f_* being the moment that catastrophic fracture of the specimen occurred and $t_{k}$ as the average value of the occurrence time of the AE hits comprising each group. In addition, the normalised applied mechanical load *ℓ* (solid red line) is depicted in the “time to failure”  time scale. At the initial loading stage, the entropic q index remained at low values (i.e., q=1.17 for *ℓ_k_* = 20%) and progressively increased alongside the applied mechanical load, attaining its maximum value q=1.41 when the normalised average applied mechanical load $\ell_{k}$ was equal to 99.8% (group 6) of the specimen’s strength. Subsequently, for the last two AE hit groups, as the average load $\ell_{k}$ exceeded 95% of the applied mechanical load, the entropic index *q* showed a slight decrease, reaching a value q=1.29 when $\ell_{k}=96.9\%$ of the applied mechanical load.

**Figure 6:**
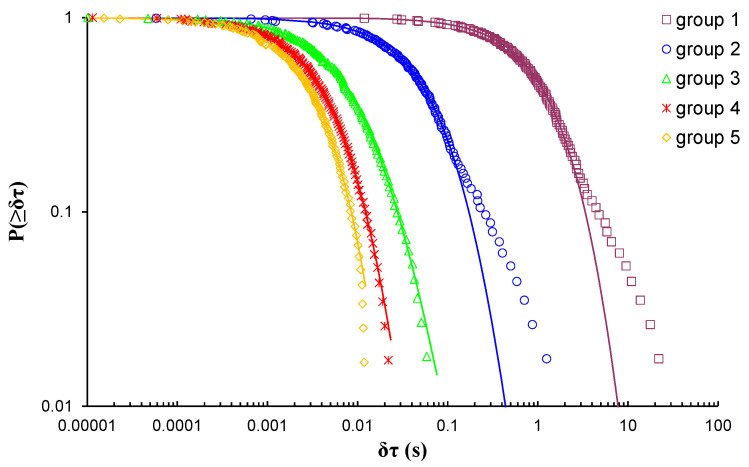
The variability of the entropic index q against the normalised average applied load $\ell_{k}$ for each AE hit group of EXP-2 in terms of the “average time before failure” parameter $\left(t_{f}-t_{k}\right)$.

**Figure 7:**
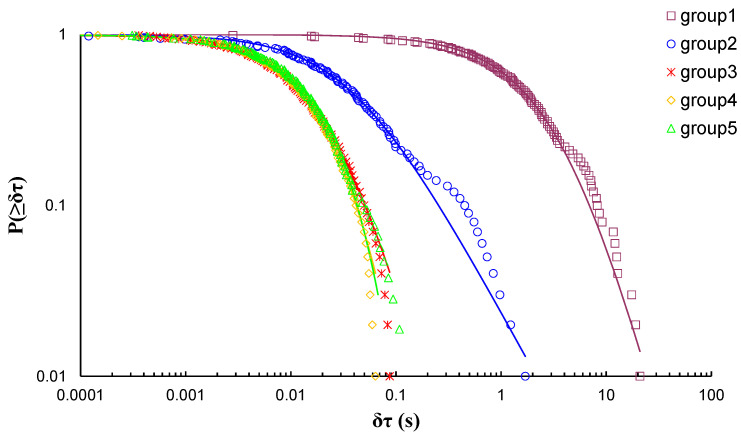
The cumulative distribution functions (CDFs) of the AE inter-event times of each AE hit group (circle markers) for EXP-3, along with the corresponding q-exponential fitting curves (solid curves).

During EXP-2, the marble specimen was subjected to direct tension. In total, five groups of AE hits were formed based on the amount of the recorded AE data, leading to the calculation of five q-indices, each of them corresponding to a different stage of the loading protocol. Figure 6 shows the log–log plot of the CDFs of the AE inter-event times *P*(>*δτ*) of each AE hit group and Table 3 presents the corresponding q-indices along the fitting parameters $\beta_{q}$ and $\tau_{q}$, which have been calculated with Equation (8), employing data points located at the initial and central areas of the CDFs, in order to avoid possible bias due to the deviations between the experimental data and the fitting model at the tails. Considering the peculiarity of the loading protocol (i.e., direct tension) the deviation of the fitting points from the experimental results, corresponding to long times *δτ*, especially at the tails of the CDFs of groups 1 and 2 in Figure 6, can be ascribed to the microcrack formation processes taking place in the bulk of the material during these stages of the loading protocol. Figure 7 depicts the temporal evolution of the calculated q indices alongside the normalised average applied mechanical load *ℓ_k_* of each AE hit group using the “average time before failure” parameter $\left(t_{f}-t_{k}\right)$ for the time scale, with $t_{f}$ being the moment when the critical fracture of the specimen occurred and $t_{k}$ as the average value of the occurrence time of the AE hits of each group. Furthermore, the normalised applied mechanical load ℓ(solid red line) is depicted in the “time to failure” $\left(t_{f}-t\right)$ time scale. During the initial stages of the loading protocol, the entropic q index began at a low value *q* = 1.12 for $\ell_{k}≃31\%$ of the applied mechanical load and progressively increased with the continuous increase in the applied load, reaching its maximum value q=1.41 when $\ell_{k}$ attained 98.4% of the applied mechanical load. A decrease in the entropic index *q* is observe during the last two AE hit groups as the average load $\ell_{k}$ exceeded 99%, reaching its lowest value q=1.09.

**Figure 8:**
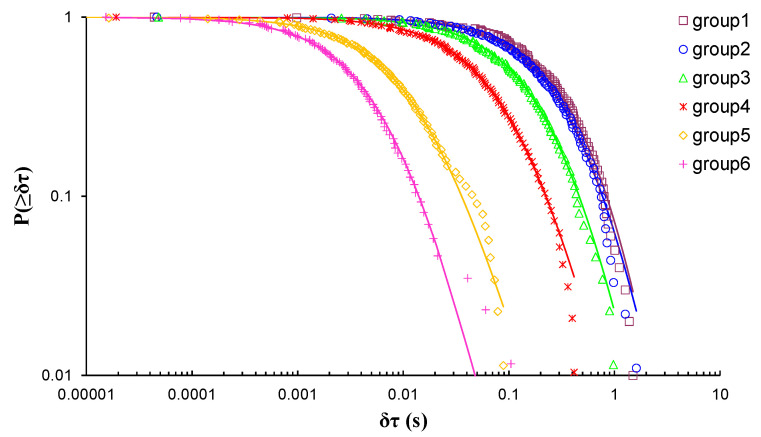
The cumulative distribution functions (CDFs) of the AE inter-event times of each AE hit group (circle markers) for EXP-4, along with the corresponding q-exponential fitting curves (solid curves).

During EXP-3, a marble specimen was subjected to diametral compression; due to the total amount of the recorded AE hits, five AE hit groups were formed resulting in the calculation of five q-indices, each of them corresponding to a different stage of the loading protocol. Figure 7 shows the log–log plot of the CDFs of the AE inter-event times *P*(>*δτ*) of each AE hit group. Table 4 presents the corresponding q-indices along with the fitting parameters $\beta_{q}$ and $\tau_{q}$, which have been calculated using Equation (8), without the data points located the tail region of all CDFs. Figure 8 presents the temporal evolution of the calculated q indices alongside the average load $\ell_{k}$ of each AE hit group using the “average time before failure” parameter $\left(t_{f}-t_{k}\right)$ for the time scale, with *t_f_* being the moment when the critical fracture of the specimen occurred and *t_k_* as the average value of the occurrence time of the AE hits of each group. In addition, the normalised applied mechanical load ℓ (solid red line) is depicted in the “time to failure” (*t_f_* − *t*) time scale. During the initial loading stages with $\ell_{k}≃79\%$ of the applied mechanical load, the entropic index *q* equalled 1.41 and increased rapidly until it attained its maximum value q=1.88 for $\ell_{k}=98\%$ of the applied mechanical load, followed by a steep decrease during the last two AE hit groups which corresponded to the fracturing region, reaching its lowest value q=1.06 at $\ell_{k}=47\%$.

**Figure 9:**
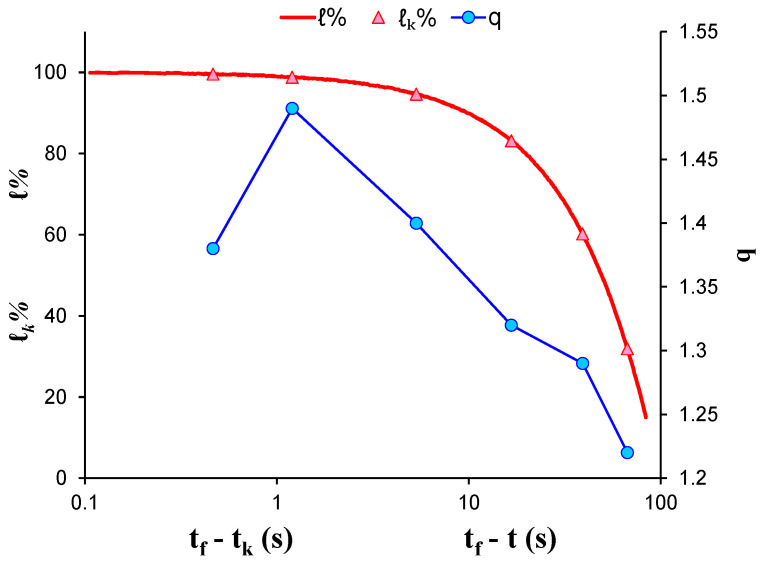
The variability of the entropic index q against the normalised average applied load $\ell_{k}$ for each AE hit group of EXP-4 in terms of the “average time before failure” parameter $\left(t_{f}-t_{k}\right)$ and the normalised applied load ℓ in “time to failure” $\left(t_{f}-t\right)$ time scale.

EXP-4 involved a cement mortar specimen that was subjected to three-point bending load. The total number of the recorded AE hits led to the formation of six AE hit groups, resulting in the calculation of six q-indices, each of them corresponding to a different stage of the loading protocol. Figure 8 shows the log–log plot of the CDFs of the AE inter-event times *P*(>*δτ*) of each AE hit group. Table 5 presents the calculated q-indices and the fitting parameters *β_q_* and $\tau_{q}$, for each AE hit group, which have been calculated using Equation (8). In order to avoid possible bias, the calculations were performed excluding the data points from the tail region of all CDFs. Figure 9 shows the temporal evolution of the calculated *q* indices alongside the average load *ℓ_k_* of each AE hit group using the “average time before failure” parameter $\left(t_{f}-t_{k}\right)$ for the time scale, with $t_{f}$ being the moment when the critical fracture of the specimen occurred and $t_{k}$ as the average value of the occurrence time of the AE hits of each group. Additionally, the normalised applied mechanical load ℓ (solid red line) is depicted in the “time to failure” $\left(t_{f}-t\right)$ time scale. A closer examination reveals a smoother increase in the entropic index q as the applied load increased in comparison with the previously described marble specimens, starting at q=1.22 for 32% of the applied load during the initial stages of the loading protocol, attaining its maximum value q=1.49 when the average applied load $\ell_{k}$ attained 98.8% of the applied load. Subsequently, for the last AE hit group, as the average applied load $\ell_{k}$ exceeded approximately 99.6% of the applied load, the entropic index q decreased to q=1.38.

**Figure 10:**
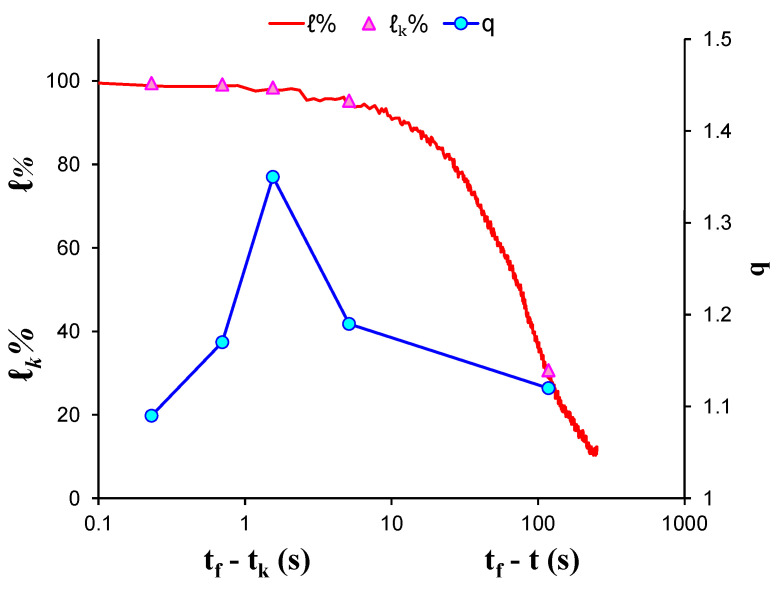
The variability of the entropic index *q* against the normalised average applied load $\ell_{k}$ for each AE hit group of EXP-2 in terms of the “average time before failure” parameter $\left(t_{f}-t_{k}\right)$ and the normalised applied load *ℓ* in “time to failure” $\left(t_{f}-t\right)$ time scale.

**Figure 11:**
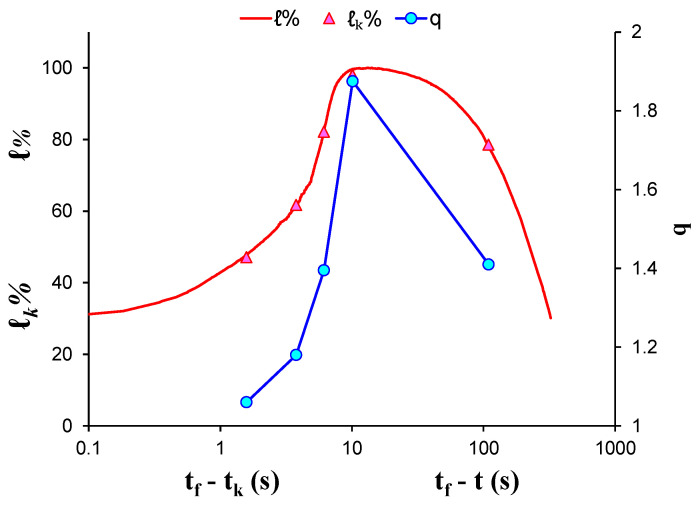
The variability of the entropic index *q* against the normalised average applied load $\ell_{k}$ for each AE hit group of EXP-3 in terms of the “average time before failure” parameter $\left(t_{f}-t_{k}\right)$ and the normalised applied load ℓ in “time to failure” $\left(t_{f}-t\right)$ time scale.

Figure 5, Figure 9, Figure 10 and Figure 11 show the evolution of the entropic q indices in regard to the normalised average applied load for each AE hit group in all cases of specimens. A closer inspection reveals a distinct behaviour where the entropic index q up to 90% of the specimens’ strength increased systematically along with the applied load, reaching a maximum value of approximately 1.35 to 1.49, apart from in EXP-3, where the maximum value of the q-index was 1.88, followed by a slight decrease during the fracturing region as the applied load exceeded 95%. The initially low values of the entropic index q can be attributed to the fact that the specimen was intact. The structure of the specimen and imperfections that are included in it existed well before the application of any external loading. Observing the values of the q index, during early loadings it becomes clear that these imperfections did not change or interact, charging the specimen bulk system. Thus, the existing imperfections were isolated in positions without spreading within the bulk material, i.e., they showed a high organisation. The continuous increase in the applied load until just before the destructive levels ($\ell_{k}$ ≤ 90%) activated the AE sources which activated the processes of creation, propagation, and development of microcracks within the specimens. The existing network of cracks began to grow even further, covering a larger volume within the specimen. Consequently, the high organisation that characterised the specimen degenerated into a less organised state; hence, the increase in the entropic index *q* and sub-additivity began to govern the system, strongly affecting the total entropy. When the load exceeded the destructive levels and the specimen entered its fracture area ($\ell_{k}\geq 95\%$), the microcracks coalesced into macrocracks, resulting in the dimensions of the macrocracks network becoming comparable to those of the specimen, and then catastrophic fracture occurs. The coalescence of microcracks into macrocracks is indicative of the self-organisation that characterises the system before breaking, hence the reduction in the entropic index q.

Special mention should be made regarding the evolution of the entropic index *q* of the EXP-2 and EXP-3 specimens (Figure 10 and Figure 11, respectively). The selection of these specific specimens was anything but random. In both cases of the specimens there was a rapid peak of the entropic index q followed by a gradual decline to its minimum value. In contrast to the other specimens (EXP-1 and EXP-4) where such behaviours were not observed (Figure 5 and Figure 9, respectively), the entropic index *q* showed a smoother transition to its maximum value, accompanied with an equally smooth one, after the maximum value of the imposed mechanical load had been reached. This can be associated to the special geometry of the specimens and the existence of tensile phenomena that appeared during their fracture, which caused the activation of the acoustic activity seconds before the failure of the specimens, thus creating two clearly separated areas of acoustic activity before and during the rupture. As mentioned, the entropic index *q* indicated randomness in the growth, spread, and gradual formation of microcracks in macrocracks. The almost instantaneous development of fractures in the EXP-2 and EXP-3 specimens, in contrast to the other specimens, translates into the behaviour of the entropic markers q shown in Figure 10 and Figure 11.

## 5. Conclusions

For the purposes of the present work, NESΜ has been employed for the analysis of AE hits recorded from marble and cement mortar specimens that were subjected to the mechanical loading of various protocols (i.e., diametral compression, three-point bending, direct tension) until fracture. The AE data that were recorded during the experiments were divided in successive AE hit groups, depending on the total amount of the recorded AE data and the normalised CDFs of the inter-event times *P*(>*δτ*) of the AE hit groups, and were plotted and fitted using exponential NESΜ modelling. The entropic index q, along with the fitting parameters $\beta_{q}$ and $\tau_{q}$, were calculated according to the Tsallis entropy model, for each AE hit group of all cases of specimens. The temporal variation of the entropic index q alongside the normalised applied mechanical load was plotted using the “average time before failure” parameter $\left(t_{f}-t_{k}\right)$ for all specimens. Results indicate a systematic relationship between the entropic index q and the applied mechanical load. Specifically, the value of the entropic index q increased progressively alongside the level of the applied load until it approached around 95% of the specimens’ strength, where the entropic index *q* attained a maximum value close to 1.35 to 1.49, except for EXP-3, where the maximum value of the *q* index was 1.88. During the last stages of the loading protocols, as the specimens entered their fracturing regions for load values ≥95% of the applied mechanical load, a steep decrease in the entropic index q appeared, followed by the catastrophic fracture of the specimens. Considering the variety of the presented experimental protocols as well as the different geometries of the studied specimens, the above findings advocate the use of the entropic index q as a potential pre-failure indicator of the upcoming failure of specimens made of brittle materials.

## Figures and Tables

**Table 1:** The implemented loading protocols, the materials of the presented specimens and the total acoustic emission (AE) hits recorded during the corresponding experiments.

Experiment	Loading Protocol	Material	Total AE Hits
1	Three-point bending	Marble	790
2	Direct tension	“	574
3	Diametral compression	“	507
4	Three-point bending	Cement mortar	549

**Table 2:** The calculated values of the entropic index *q*, along with the calculated fitting parameters $\beta_{q}$ and $\tau_{q}$, for the specimen in EXP-1.

AE Hit Group	$\left(t_{f}-t_{k}\right)\left(s\right)$	q	$\beta_{q}\left(1/s\right)$	$\tau_{q}\left(s\right)$	$\ell_{k}$ (%)
1	881.12	1.17	0.24	4.167	19.8
2	490.93	1.21	0.32	3.125	50.7
3	253.75	1.19	0.75	1.333	74.2
4	124.65	1.27	1.10	0.909	88.5
5	44.70	1.38	2.50	0.400	97.3
6	8.65	1.41	10.50	0.095	99.8
7	2.59	1.35	94.90	0.011	98.7
8	1.06	1.29	100.10	0.010	96.9

**Table 3:** The calculated values of the entropic index q, along with the calculated fitting parameters $\beta_{q}$ and $\tau_{q}$, for the specimen in EXP-2.

AE Hit Group	$\left(t_{f}-t_{k}\right)\left(s\right)$	*q*	$\beta_{q}\left(1/s\right)$	$\tau_{q}\left(s\right)$	$\ell_{k}$ (%)
1	117.8	1.12	0.79	1.2658	30.7%
2	5.13	1.19	16.8	0.0595	95.3%
3	1.55	1.35	127.8	0.0078	98.4%
4	0.70	1.17	230.2	0.0043	99.2%
5	0.23	1.09	300.5	0.0033	99.6%

**Table 4:** The calculated values of the entropic index q, along with the calculated fitting parameters $\beta_{q}$ and $\tau_{q}$, for the specimen in EXP-3.

AE Hit Group	$\left(t_{f}-t_{k}\right)\left(s\right)$	*q*	$\beta_{q}\left(1/s\right)$	$\tau_{q}\left(s\right)$	$\ell_{k}\;\left(\%\right)$
1	109.27	1.41	0.55	1.803	78.5%
2	10.12	1.88	29.26	0.034	98.0%
3	6.12	1.40	74.00	0.014	82.1%
4	3.77	1.18	68.11	0.015	61.7%
5	1.58	1.06	57.78	0.017	47.1%

**Table 5:** The calculated values of the entropic index q, along with the calculated fitting parameters $\beta_{q}$ and, for the specimen in EXP-4.

AE Hit Group	$\left(t_{f}-t_{k}\right)$ (s)	*q*	$\beta_{q}\left(1/s\right)$	$\tau_{q}\left(s\right)$	$\ell_{k}\;\left(\%\right)$
1	67.07	1.22	3.56	0.2809	31.9%
2	39.22	1.29	4.25	0.2352	60.2%
3	16.68	1.32	7.34	0.1362	83.2%
4	5.33	1.40	16.86	0.0593	94.6%
5	1.20	1.49	119.4	0.0084	98.8%
6	0.47	1.38	262.4	0.0038	99.6%

## Data Availability

Not applicable.
